# Prevalence and Associated Factors of Erectile Dysfunction among Married Men in Vietnam

**DOI:** 10.3389/fpubh.2017.00094

**Published:** 2017-05-04

**Authors:** Thang Van Vo, Hue Dinh Hoang, Nhan Phuc Thanh Nguyen

**Affiliations:** ^1^Institute for Community Health Research, Hue University of Medicine and Pharmacy, Hue, Vietnam; ^2^Faculty of Public Health, Hue University of Medicine and Pharmacy, Hue, Vietnam

**Keywords:** erectile dysfunction, married men, depression, quality of life, sexual health

## Abstract

**Background:**

Sexuality is an essential part of life; however, erectile dysfunction (ED) has been one of the most common complaints among men with sexual health issues all over the world. ED includes dysfunction in erection and penile erectile pain. In Vietnam, ED is a subject a not readily discussed. Thus, relatively little is known about ED among Vietnamese men.

**Aims:**

To identify the prevalence of ED and its associated variables and the need for treatment of ED among married men in Vietnam.

**Methods:**

This was a cross-sectional study. A total sample size included 746 married men, aged 20–60 years, living in four representative wards of the Hue City and randomly selected by systematic sampling methods. Respondents completed a self-reported questionnaire. The International Index of Erectile Function (IIEF-5) scale was used to determine ED severity, and the Depression Anxiety Stress Scales (DASS-21) was used to measure depression, anxiety, and stress. Quality of life was assessed using the WHO Quality of Life score (WHOQoL). A multivariate logistic regression model was used to determine the relationships between independent variables and ED.

**Results:**

Mean age of married men was 44.3 ± 8.7. Two-thirds (66.9%) of respondents experienced ED symptoms. In terms of severity, 40.8% reported mild ED; 20.3% mild–moderate ED; 5.0% moderate ED; and 0.8% severe ED. Depression, anxiety, and stress problems were 5.0, 3.6, and 2.8%, respectively. One-third (33.1%) of the respondents reported having low quality of life, and 32.6% reported having medium quality of life. The vast majority (86.9%) had consensual sex with their wives/partners. Variables associated with increased IIEF-5 score were increased WHOQoL score, increased body mass index (BMI), religion, and no consumption of alcohol. Increasing age, disease history, increased anxiety, and no consensual sex with their wife/partner were associated with a lower IIEF-5 score. If experiencing ED, 55.5% would seek help from medical doctors, 55.1% discussed it with their wives/partners, and 23.1% turned to their friends for help.

**Conclusion:**

The prevalence of ED was high, although only 5.8% experienced moderate to severe ED. The key factors associated with ED were age, religion, disease history, BMI, alcohol consumption, anxiety, quality of life, and consensual sex with their wives/partners. Sexual health education should be more specifically targeted for men, including the provision of local sexual health-care services for men.

## Introduction

Sexuality is an essential part of life, however, erectile dysfunction (ED) has been one of the most common complaints among men with sexual health issues (1). ED includes dysfunction in erection and penile erectile pain. Research on this subject typically only discusses decreased ED issues (also called impotence). ED is a subject not readily discussed in Vietnam, and thus, little is known about ED among Vietnamese men. This study sought to document the prevalence of ED among married men in Vietnam and to explore care options.

The Massachusetts Male Aging Research Study (MMAS) on ED prevalence, conducted in 1999, suggested that ED may affect 152 million men worldwide and estimated that this figure would increase to 322 million by 2025 (2). Other research, such as Selvin et al. (3) in the USA, showed the proportion of people over 20 years of age with ED to be 18.4%. The study also indicated that ED affected about 18 million men in the USA (3). In short, ED is a common health concern worldwide (4). ED is significantly associated with aging and other common non-communicable diseases (NCDs), such as cardiovascular disease, diabetes, mental health disorders, trauma, and interpersonal problems. Unhealthy lifestyle also contributes to problems with ED (5).

In Vietnam, ED research is mostly done in urology clinics and in hospitals. In 1997, Pham’s study conducted in four different geographical and economic regions (Hai Duong, Nam Ha, Hanoi, and Dong Thap) showed the prevalence of ED in the aggregate to be 15.7%; specifically, 10.8% among men aged 18–30, 44.0% among those aged 40–45, and 57.0% for men aged 60 and above (6). However, this research had its limitations and was not able to identify any causal factors to link ED to life style and/or chronic medical conditions. Vietnam has very limited sexual education, counseling, and treatment services, especially aimed at men having ED and their need to access adequate care. Therefore, it is essential to have more studies on ED prevalence and possible causes as recognized epidemiologically to be psychogenic and multifactorial in order to fill the gaps in our knowledge of ED and its risk modification.

Sexual perception and sexual disorders are seriously affected by traditions and cultural factors of the local people. Hue city was the ancient capital of Vietnam (1802–1945) under the former Nguyen dynasty, where different value systems coexisted: the Confucian social and moral ethics; Champa civilization; and Western influences. Gender role in Confucianism with men being “superiors” and women as “subordinates” is still prevalent at the present time. ED, therefore, is not only a sensitive and taboo subject but also a health problem that men are often embarrassed and scared to share while they actually want to have direct access to local friendly health services for treatment. In fact, this kind of sexual health service does not exist in Vietnam, and the number of experts who can provide ED consultancy and treatment is quite limited. As a result, Hue city was chosen as the most representative setting in Vietnam for this study to measure ED prevalence and associated factors as well as to identify the need among married men for treatment of ED.

## Methodology

A cross-sectional survey was conducted among representative married men, aged 20–60 years, in Hue city, Vietnam, from March through September, 2015.

### Sample Size

The formula $n=Z_{1-\alpha /2}^{2}\frac{(1-p)p}{\varepsilon^{2}}$ was used to calculate desired sample size as follows: $Z_{1-\alpha /2}^{2}$: reliability coefficient with α = 0.05, so $Z_{1-\alpha /2}^{2}={\left(1.96\right)}^{2}$; *p* = 0.157 based on ED ratio of Pham (6); ε: relative error, ε = 0.05; therefore: *n* = 204.

The sample size was multiplied by a design factor of 2, plus 10% reserved sample size. A total of 450 people were needed for the study. Multi-stage cluster sampling was used to select 746 subjects living in 746 randomly selected households at hamlet level, this being the smallest administrative unit in Hue City, from the sample frame provided by communal health centers.

Subjects were identified by using a systematic sampling of households in four representative wards of the city. A list of households was supplied prior to the survey, by the local National Population and Family Planning Management Program. Household was the basic sampling unit with one subject each as observation unit got chosen at random. Sample members were selected according to a random starting point and a fixed periodic interval. This sampling interval was calculated by dividing the population size of married men (*N* = 6,730) by the desired sample size (*n* = 746).

Data collectors responsible for household listings were local population collaborators with sufficient awareness of the geographical characteristics and population distribution within the enumeration area. Subjects were asked to complete an informed consent form, and confidentiality of all data was assured. Ninety-eight percent (98.0%) of those invited, agreed to participate in the study. Reasons for decline included poor health, illiteracy, and refusal of being interviewed.

### Data Collection Methods

Those agreeing to participate in the study were interviewed by selected male family planning collaborators. Participants were requested to complete the self-reported questionnaire of IIEF-5 in the privacy of their own homes and to return it upon completion of the interview. Guidance was provided to respondents, especially illiterate men, who needed assistance to complete the self-reported questionnaires.

### Research Contents

○ The questionnaire had five main components, the majority of which had been validated and used in other studies. The general information section included age, education, religion, occupation, income status, family composition, marital status, and living situation.
○ Height and weight were self-reported, which were then converted to a measure of body mass index (BMI).
○ Disease history: NCD (10 medical records through self-reporting and additional health records, drug medication side effects, disease lasting from 6 months and longer including arthritis, asthma, cancer, chronic bronchitis, diabetes, heart disease, hypertension, stroke/vascular sclerosis, osteoporosis, and trauma) and others.
○ Depression disorders, anxiety, and stress: the Depression Anxiety Stress Scales with 21 items (DASS-21) (Lovibond and Osman) was used ([ref]^7^, ^8^, ^10^). They were classified as follows:

**Table:** 

Items	Normal	Mild	Moderate	Severe	Very severe
Depression	0–9	10–13	14–20	21–27	28+
Anxiety	0–7	8–9	10–14	15–19	20+
Stress	0–14	15–18	19–25	26–33	34+
Healthy—happiness index and quality of life, including health assessment: this element was measured using questions from three validated instruments: the WHO-5 (WHO-5 Well-being Index) scale; 4-item Subjective Happiness Scale to evaluate levels of happiness (scale 1–7); the WHO Quality of Life score BREF scale for evaluating quality of life (categorized into four main fields: physical, mental, social, and environmental). WHO-5 was a convenient instrument, has been used in mental health research worldwide, and has a good psychometric validity for measuring the well-being index. In Vietnam, these scales have been translated into Vietnamese and validated in previous studies of youth mental health (10). Domain score was scaled in a positive direction, higher scores denote higher quality of life. In this research, we determined people who had a total score higher than 66.67% to be in the “high quality of life” group.
ED: IIEF-5 was used to determine the status of ED. This scale in its Vietnamese version was validated in a recent study in Vietnam (11). In our ED research, we used IIEF-5 to assess ED. The tool was pretested with local people to adjust wording before data collection. In addition, Cronbach’s alpha index of IIEF-5 was calculated in our pilot research as >0.7, and thus had a good reliability for a psychometric test.

To assess ED level, respondents filled out the IIEF questionnaire. This is a shortened screening scale with 15 questions with 5 themes related to ED (erections and orgasms, sexual desire, satisfaction with intercourse, and agreed sexual contentment) developed by the National Institutes of Health of the USA. The IIEF scale has five assessment questions about men’s sexual life across different cultures. Responses are scaled using a Likert scale from 0 to 5. Total score range from 1 to 25. The cutoff point for identifying ED is a score of 22 or above as noted below (12):

○ No ED: 22–25
○ Mild: 17–21
○ Mild–moderate: 12–16
○ Moderate: 8–11
○ Severe: 1–7.

For correlation analysis, the variable for ED was divided into two groups:

- ED if total score: ≤21
- No ED: if total score: >21.

### Data Analysis

SPSS software was used for data processing. Descriptive statistical analysis (frequency and percentage) were calculated for all variables, and χ^2^ test at α = 0.05 significance was used to compare the difference between two or more odds ratios. A multivariate logistic regression analysis to determine the associations between the independent variables and ED was carried out for the variables that were significant in the bivariate analysis. Stepwise backward method was used in the multivariate regression model. Statistical significance was defined as *p* < 0.05 for the independent variables in the final model.

## Results

Demographic information of respondents is reported in Table 1 below.

**Table 1:** **Demographic information of respondents (*n* = 746)**.

Variables			Frequency	Percentage
**Age groups (in years)**	20–29		41	5.5

	30–39		184	24.7

	40–49		289	38.7

	50–60		232	31.1

Mean			44.3 ± 8.7	

Median			45	

**Education**	Illiterate		7	0.9

	Primary and secondary		328	44.0

	High school		245	32.8

	Intermediate and college		70	9.4

	Undergraduate and postgraduate		96	12.9

**Occupation**	Civil servants		143	19.2

	Others		603	80.8

**Religion**	Yes	Buddhist	390	52.3

		Christian	51	6.8

	No		305	40.9

**Economic situation**	High		131	17.6
	Median		601	80.6

	Poor		14	1.8

**Total**			746	100.0

### Lifestyle Behaviors

Respondents were asked about smoking, alcohol consumption, and exercise. Nearly 60% (58.4%) reported smoking and 24.3% as heavy smokers who smoked more than 10 cigarettes per day.

Alcohol consumption included 70.8% of beer; 16.0% of other alcoholic drinks; and 26.4% of a combination of beer and other alcoholic beverages. Nonetheless, 54.9% drank less than two cans of beer per day, and 86.6% drank less than 60 ml of alcohol per day.

About one-third of the respondents (33.5%) engaged in physical activities, and nearly two-thirds (62.0%) of them did so on a regular basis. The primary activity (66.8%) was walking.

### Health, Mental Health, Quality of Life

The average BMI index was 21.94 ± 2.11, with two-thirds of the respondents being in a normal range, 27.9% being overweight, and 4.7% being underweight.

Regarding disease history, 13.8% of respondents reported having been diagnosed with chronic diseases and other health problems. Of these, 66.0% were diagnosed by governmental health services and the rest (34.0%) were diagnosed by a private health service and other medical providers.

Mental health symptoms were relatively low in prevalence. Only 5.0% of the respondents reported having symptoms of depression, 3.6% reported anxiety, and 2.8% reported stress.

With regard to quality of life, 70.2% stated they had a high quality of life, 42.1% had higher than the average level, and 69.1% were satisfied with their health. The average score for quality of life was 63.73 ± 10.40.

### Prevalence of ED

According to self-reported responses to the questions of the IIEF-5 scale, 499 respondents (66.9%) had scores ≤21, that is, those with signs of ED.

Table 2 shows the distribution of ED levels among the respondents. About two-thirds of the respondents (66.9%) reported having signs of ED, most at a mild level (40.8%) and only 0.8% at a severe level of ED.

**Table 2:** **Distribution of erectile dysfunction (ED) levels**.

ED level	IIEF-5 score	*N*	Percent
Non ED	22–25	247	33.1
Mild ED	17–21	304	40.8
Mild–moderate	12–16	152	20.3
Moderate	8–11	37	5.0
Severe	1–7	6	0.8
Total		746	100.0

### Associated Variables with ED

When considering the independent variables in bivariate analyses, as seen as the crude OR results in Table 3, the prevalence of ED in this study showed a statistically significant association with age groups and also with occupation, religion, BMI, alcohol/beer consumption, anxiety, quality of life, average number of hours of sleeping, and consensual sex with their wife/partner during sexual activity. No association was found between ED with education, economic situation, disease history, tobacco use, and/or physical activity.

**Table 3:** **Multivariate logistic regression model**.

Variables		Crude OR (95% CI)	Adjusted OR (95%CI)
Age groups	20–29	1	
	30–39	1.66 (0.84–3.29)	1.94 (0.94–4.00)
	40–49	2.45** (1.26–4.76)	2.89** (1.42–5.89)
	50–60	5.31** (2.64–10.66)	5.77** (2.71–12.27)

Occupation	Civil servants	1	–
	Others	1.50* (1.03–2.18)	–

Religion	No	1	1
	Yes	0.69* (0.50–0.94)	0.57** (0.40–0.80)

Disease history	No	1	1
	Yes	2.82** (1.63–4.86)	1.95* (1.09–3.48)

Body mass index	≥23.0	1	1
	<18.5	2.48* (1.07–5.71)	2.58* (1.04–6.37)
	18.5–22.9	1.71** (1.22–2.39)	1.70** (1.18–2.46)

Alcohol consumption	No	1	1
	Yes	1.81** (1.30–2.54)	1.82** (1.26–2.64)

Anxiety	No	1	1
	Yes	6.46** (1.52–27.50)	3.92 (0.85–18.02)

Quality of life	High	1	1
	Low and middle	3.21** (2.17–4.74)	2.24** (1.47–3.40)

Consensual sex with wife/partner	Yes	1	1
	No	2.10** (1.25–3.53)	1.80* (1.03–3.15)

Hours sleeping	≥7 h	1	–
	<7 h	2.01** (1.47–2.73)	–

*Statistically significant at p < 0.05 level of confidence.

**Statistically significant at p < 0.01 level of confidence.

The model of multivariate logistic regression was also applied, adjusting for the OR to control confounding factors as shown in the Table 3.

Cofactors associated with ED are age, religion, disease history, BMI, alcohol consumption, anxiety, quality of life, and consensual sex with their wives/partners (*p* < 0.05).

Health seeking behavior in ED revealed an actual need for treatment among married men with 55.5% opting for medical help and doctors, 55.1% discussing the problem with their wives/partners, and 23.1% sharing the problem with their friends for help.

## Discussion

Two-thirds (66.9%) of married men had signs of ED. This rate is higher than previously reported by other studies. However, variations in samples and study design could account for the differences in ED prevalence result.

According to the most recent report by Park et al. (13), the rate of ED for men from Asian countries ranged from 2.0 to 88.0% (13). The average rates in each age group were 15.0% for 20–29, 30.0% for 30–39, 41.0% for 40–49, 54.0% for 50–59, and 70.0% for 60–69. According to the research by Shaeer et al. (14), the ED rate among men was 57.4% in Nigeria, 63.6% in Egypt, and 80.8% in Pakistan (14). Our study reported a rate just lower than Pakistan’s.

In Vietnam, Pham studied married men from 20 to 60 years old and found an overall ED rate was 15.7% (6). The rate found in our current study is considerably higher. However, it was lower than the result of 83.7% found by Vo et al. (11) in Ho Chi Minh city. It is worth noting that Vo et al.’s research sample was men between 30 and 65 of age with type II diabetes (11).

In analyzing factors influencing ED in our study, some interesting findings were found. This allowed us to make meaningful comparisons between our study and others reported in the literature, as follows:

### Age

In our study, respondents in the eldest age group (50–60 years) had a 5.77-fold higher risk of experiencing ED than respondents in the youngest age group (20–29 years). Across the six countries, ED prevalence rates increased from 4.0–6.0% in men younger than age 40 years to 39.0–73.0% in respondents aged 70–75 years. These findings are consistent with those from another population-based study. Kinsey stated that aging is an important risk factor for the development of ED in men. Kinsey’s research showed that the proportion of ED increased with age from 0.1% at 20 years old to 75.0% at 80 years old (15). The results of the MMAS showed the same trend with the prevalence of ED of 39.0% in men aged 40 increasing to 67.0% for men in their 70s (16). In the study reported here, age had a positive association with the rate of ED. Among men age 20–29, the rate of ED was 5.5%. Among men age 30–39, the rate was 24.7%. The rate of ED for men in their 40s was slightly more than for men in their 50s, but both were in the 30–40% range, at 38.7 and 31.1%, respectively. Age is a risk factor for ED, with ORs statistically significant with age: age group 40–49 had 2.89 times at risk of ED (CI: 1.42–5.89) compared to those 20–29 years old; age group 50–60 had a 5.77 times higher risk (CI: 2.71–12.27) compared to the 20–29 age group. The risk difference between those in age group 39–39 and those age 20–29 was not significant (*p* = 0.15 > 0.05). The results found in our study were similar to the conclusion from a 2007 meta-analysis by Cheng of studies in Asian countries that the rate of ED increased with age (17).

Pham’s research study of 764 married men showed an ED rate of 10.8% in the 18–30 age group; 44.0% in the 40–45 age group; and 57% in the 60 and above age group (6). Other research analysis indicated a significant positive correlation between ED and increasingly poor health. Tuan’s study of diabetic patients also showed increased ED with age and this disease severity.

### Occupation

Occupation was simply coded into two categories of civil servants and non-civil servants. The percentage of occupation as civil servants was 19.2%. In other similar community-based research, the effect of occupation remained statistically significant to ED, especially for men who perform manual labor tasks. Those in manual labor were significantly more likely to develop ED (18). In our study, occupation of respondents also displayed a relationship with ED with bivariate analysis but this association did not remain when multivariate logistic regression model was applied.

### Religion

Married men who followed a religion were 0.59 times more likely to report ED than those who reported not to follow any religion (CI: 0.40–0.80). This finding was quite different from Canadian Sexuality Education Resource Centre, which mentioned that in some cases, negative attitudes about sex in some strict religious beliefs could create sexual guilt or anxiety surrounding intercourse (19).

### Disease History

Respondents were asked to self-report on 10 different chronic conditions, if existed, in their disease history. The highest prevalence was arthritis (4.2% of respondents), followed by hypertension (1.6%), and injuries (0.9%). Our study showed ED was significantly higher in respondents with disease history (OR = 1.95, CI: 1.09–3.48). The most important organic causes of ED were cardiovascular disease and diabetes, among other health problems. Moreira’s research conducted in Salvador showed 602 respondents in the study had a history of common diseases with hypertension at 24.9%, heart disease at 7.7%, and depression at 5.8%. Seftel et al. (20) showed that hypertension, hyperlipidemia, diabetes, and depression were common in men and related to ED. Cho et al. (21) also concluded ED rates in Korea were significantly higher in patients with chronic diseases, such as diabetes, depression, and anxiety. The incidence of ED among men with high blood pressure was lower than people with diabetes or with both conditions (21).

### Body Mass Index

Our results showed that those with low BMI were at 2.58 times higher risk for ED than those with a BMI of 23.0 or higher (CI: 1.04–6.37). There were different findings from other research that supported our study, for example, the recent results by Weber et al. (22) in Australia indicated that BMI was associated with ED in obese respondents (22). Cho’s research in 2003, however, did not find any association between obesity and increased risk of ED among Korean men (21).

### Lifestyle Habits

#### Smoking

Our study found no significant association between the use of tobacco and ED. Moreira et al. (23) showed that smoking (current or former) was not associated with ED and concluded that the association between smoking and current ED was difficult to detect in cross-sectional studies (23). However, Mannino et al.’s (24) study on men in the USA showed an association between ED and smoking (OR = 1.8; CI: 1.2–2.6) (24). In contrast, Bai et al. (25) showed that smoking was not related to ED but duration of tobacco use was related to ED (25).

A more thorough investigation about smoking (specifically, the effect of nicotine), daily consumption volume, smoking duration, existence of cardiovascular disease, as well as other chronic diseases, could allow us to gain a more accurate understanding of the relationship between tobacco use and ED.

#### Beer/Alcohol Consumption

Vietnam is a country with a high rate of alcohol consumption. In our study, 26.4% of respondents reported drinking both alcohol and beer, 70.8% just beer, and 16.0% taking other alcoholic beverages. More than half (54.9%) drank less than two cans of beer/day, and 86.6% drink less than 60 ml of alcohol/day. The risk of ED for frequent drinkers was 1.82 times compared to non-frequent ones (CI: 1.26–2.64). This result was similar to Moreira et al.’s (23) research and showed an inversely proportional relationship between alcohol consumption in moderation and ED in multivariate analysis (23). Weber at al.’s (22) study in Australia showed that alcohol consumption in moderate quantity significantly reduced the risk of ED among men in the age group 45–54 but not in older age groups (22). This result was similar to Moreira’s study. According to Bai et al. (25), drinking alcohol was inversely related with ED (*p* < 0.01), but drinking duration was directly proportional to the rate of ED (*p* < 0.01) (25).

#### Physical Activity

Only one-third (33.5%) of respondents engaged in some form of physical activity, including walking, playing badminton, soccer, and various other sports. Just under two-third (62.0%) engaged in regular activities (5 or more days/week), and 36.8% in less regular (2–4 days/week). However, the study found no statistically significant association between physical activity and ED.

### Depression, Anxiety, and Stress

Psychological factors strongly related to ED have been shown in the medical literature. In this study, 5.0% of respondents reported having depression; 3.6% suffering from anxiety, and 2.8% stress. The comorbidity rate in our respondents with ED is high: 78.4% with depression, 81.0% with stress, and 92.6% with anxiety. However, only respondents with anxiety showed a statistically significant relationship with ED (adjusted OR = 3.92). This result was not consistent to the studies of Cho et al., Cheng et al., and Seftel et al., all of which showed a relationship between ED and depression (17, 20, 21).

### Quality of Life

The impact of ED on QoL has become very important in the management of ED. Most of the medical treatments for ED up until now focused on improving QoL of patients (26). Mean score of QoL in this study was 63.73 ± 10.40. The highest average score was 72.66 ± 12.95 in the physical dimension. The lowest average score was 56.04 ± 12.80 in the environmental dimension. More than four-fifth (82.9%) of those reporting ED also reported having a low or moderate quality of life, whereas 60.1% of those with no ED reporting having high quality of life. The low and middle quality of life group was 2.24 times more likely to report ED compared to those scoring high on the quality of life scale (CI: 1.47–3.40).

Mental health variables surprisingly did not show significant relationships with ED, except for anxiety. However, quality of life variables did show an association. Further research into the mental and emotional aspects of life that could be related to ED and the direction of causality would be useful to guide future education and treatment programs. For example, did low quality of life result in ED or did ED result in low quality of life? These interactions remained to be examined, not only in Vietnam, but for prevention, diagnosis, and treatment of ED worldwide.

### Consensual Sex with Their Wives/Partners

More than three-fourths of the respondents (78.6%) reported having had sex in the past 4 weeks, and 86.9% reported having consensual sex with their wives/partners. Those without consensual sex with their wife/partner had 1.80 times higher risk for ED than those who did report consensual sex (CI: 1.03–3.15).

### Average Amount of Sleep Each Night

The average amount of sleep each night of the respondents was 7.25 ± 1.04 h (Min: 3–Max: 10), which is in the normal range for adults. The average amount of sleep for those who reported ED is less than the average (*p* < 0.000). But this statistically significant relationship did not remain when multivariate logistic regression model was applied (see Table 3).

### Education

In our study, the analysis did not reveal a statistically significant relation between education and ED. Similarly, in a study of three major cities in China, Bai et al. (25) showed a univariate association between education level and ED (25); however, this study did not find any association with statistical significance between general education and ED.

Crude OR was obtained as seen in Table 3. The results of logistic regression analysis showed 10 independent variables with a statistically significant association (*p* < 0.05) to ED to include age, occupation, religion, disease history, BMI, alcohol consumption, anxiety, quality of life, consensual sex with their wife/partner, and number of hours of sleep per day.

Adjusted OR was taken into account the effect due to all the additional independent variables included in the analysis to control for confounder variables for the said relationship, followed by multivariate regression logistic analysis substantiated that only eight independent variables had a statistically significant relation with ED. These included age, religion, disease history, BMI, alcohol/beer consumption, anxiety, quality of life, and consensual sex with their wives/partners during sexual activity. Mental health variables did not indicate strong associations with ED, but this could be attributed to the very low levels of mental health problems reported by the respondents.

The detailed analysis reported above confirmed the findings of this study to be, for the most part, consistent with similar studies of ED conducted in other countries and regions. The relationship of ED with certain variables, for example, smoking, remains unclear despite several studies and demands for more refined research.

A study on male sexual dysfunction in Asia by Ho et al. showed that, in contrast to women, men are less likely to comply with advice from a health service consultant (27). Results of the study reported here have clear implications for actions, based on our findings on health-seeking behavior among married men in ED treatment, with the rather high rate of 55.5% respondents expected to see medical doctor. This finding was in line with many recent studies. Sexual awareness and positive attitudes of men toward ED diagnosis and treatment-seeking behaviors have improved, especially in Asian countries. Our study expected to make a considerable contribution to clarification of the role of local health services in meeting the need of community-based sexual health care for men.

The study has several limitations. The sample of respondents came from residents of Hue city in Central Vietnam. A sample covering a broader area, including rural villages, would serve to reinforce or refine the findings. The study examined associations between independent variables and ED but was limited in showing causation. Moreover, chronic illness and the other health problems among the respondents were self-reported. A clinical study done within the medical system might elucidate the relationship between ED and various physical conditions.

## Conclusion

The research provided very clear evidence of the high prevalence of ED among married men. ED showed a statistically significant relationship with age, religion, disease history, BMI, alcohol consumption, anxiety, quality of life, and having consensual sex with their wives/partners. This study was also the first community-based survey in Vietnam and provided strong evidence of the need for health-related actions pertaining to ED for married men.

Health education for men, women, and families should be conducted in the community as well as through health centers and hospitals. A number of techniques and messages are available for health education purposes, and it should be possible to select those that are suitable and effective for the Vietnamese population. Overcoming any embarrassment or stigma felt by men and/or their wives is a first step in encouraging men to seek diagnosis and treatment.

Community-wide health education should be offered to improve knowledge about erectile and sexual dysfunction to avoid undue anxiety and to encourage men to consult medical professionals regarding sexual function concerns. An essential strategy is to integrate and deploy consultant services in reproductive health in general, and men’s sexual health in particular, by screening for early detection and treatment. In addition, men should be encouraged to engage in a healthy lifestyle with regular physical activity and less alcohol use.

The results have striking implications for action by health-care providers. The high prevalence of ED, with about two-thirds of the respondents reporting some level of ED, indicates a need for the formal health-care services to pay more attention to men’s sexual health. Questions about and diagnosis of sexual functioning should be incorporated into visits by men to health centers and hospitals. Physicians should be trained to ask appropriate questions of male patients, despite the sensitive nature of sexual conditions. The respondents in this study were clearly willing to report problems with sexual functioning on a questionnaire, so practitioners could expect at least a fair degree of honesty in responding to questions asked during a medical examination.

In summary, this study contributes to the understanding of the prevalence of ED among men in Vietnam. Further studies should include a broader sample geographically and extend beyond men who are currently married. Data can provide guidance for effective education, prevention, and treatment programs to address the challenges of ED in Vietnam and worldwide.

## Ethics Statement

This is a study related to sexuality and conducted in a gender-sensitive community. Thus, it was necessary to ensure compliance with three principles of research ethics: respect for privacy, fairness, and benefit to the respondents. Prior to each interview, participants were clearly advised of the research purposes and that participation was entirely up to them. The respondents were assured that they could stop at any point, if they so desired, and that their responses would be kept completely confidential, as all data would be full encrypted. The benefits to the respondents were explained as the research results would be used as the basis for planning interventions for prevention and treatment of erectile dysfunction, as well as development of local programs for sexual health care with the objective of enhancing the quality of life for men in Hue City in particular and in Vietnam in general. This study was submitted to and approved by the Institutional Review Board of Hue University of Medicine and Pharmacy.

## Author Contributions

TV contributed to the first draft of the manuscript including data collection process management and data analysis. HH and NN were responsible for doing basic steps in data collection and analysis. HH also was responsible for checking the reliability of data set as well as performing further data analysis using multivariate models. All authors had a strong consensus and approved the manuscript before it submitted to the Frontiers Journal.

## Conflict of Interest Statement

The authors declare that the research was conducted in the absence of any commercial or financial relationships that could be construed as a potential conflict of interest.
